# Enhanced production of tanshinone IIA in endophytic fungi *Emericella foeniculicola* by genome shuffling

**DOI:** 10.1080/13880209.2018.1481108

**Published:** 2018-09-28

**Authors:** Pengyu Zhang, Yiting Lee, Xiying Wei, Jinlan Wu, Qingmei Liu, Shanning Wan

**Affiliations:** College of Life Sciences, Shaanxi Normal University, Chang’an Campus, Xi’an, Shaanxi, China

**Keywords:** Gene recombination, strain breeding, metabolic production, RAPD

## Abstract

**Context:** Tanshinone IIA, commercially produced from *Salvia miltiorrhiza* Bunge (C.Y.Wu) (Labiatae), has various biological benefits. Currently, this compound is mainly extracted from plants. However, because of the long growth cycle and the unstable quality of plants, the market demands can barely be satisfied.

**Objective:** The genomic shuffling technology is applied to screen the high-yield tanshinone IIA strain, which could be used to replace the plant *S. miltiorrhiza* for the production of tanshinone IIA. The change in the production of tanshinone IIA is clarified by comparing it with the original strain.

**Materials and methods:** Tanshinone IIA was extracted from Strains cells, which was prepared through 0.5 mL protoplast samples by using hypertonic solution I from two different strains. Then, it was analyzed by high-performance liquid chromatography at 30 °C and UV 270 nm. Total DNA from the strains was extracted for RAPD amplification and electrophoresis to isolate the product.

**Results:** In this study, a high-yield tanshinone IIA strain F-3.4 was screened and the yield of tanshinone IIA was increased by 387.56 ± 0.02 mg/g, 11.07 times higher than that of the original strain TR21.

**Discussion:** This study shows that the genetic basis of high-yield strains is achieved through genome shuffling, which proves that genome shuffling can shorten the breeding cycle and improve the mutagenesis efficiency in obtaining the strains with good traits and it is a useful method for the molecular breeding of industrial strains.

## Introduction

Over the past several years, tanshinone IIA has been commercially produced from the dried roots and rhizomes of *Salvia miltiorrhiza* Bunge (C.Y.Wu) (Labiatae) (i.e. Tan-Shen in Chinese), a well-known traditional Chinese herbal medicine. In recent years, its various biological benefits have been found, such as the ability of promoting blood circulation and treating hemorrhages, menstrual disorders and miscarriages (Lee et al. 1987; Xu 1990).

It also has some inhibitory capabilities including antioxidant, antithrombosis, antihypertension, antitumor, etc. (Jiang et al. 2005). Unfortunately, the natural medicinal plants of tanshinone IIA are currently in short supply because of the over collection of the wild plants and environmental change (Kang et al. 2003). Many approaches have been applied to enhance the production of tanshinones from *S. miltiorrhiza*, including genetic transformation (Yan and Wang 2007), Ag^+^ elicitation (Zhang et al. 2004), β-aminobutyric acid induction (Ge and Wu 2005), *in situ* adsorption and semi-continuous operation (Yan et al. 2005). To provide an alternative source of natural tanshinone IIA, the endophytic fungus TR21 was isolated, and it contained a certain amount of tanshinone IIA (Wei et al. 2010). Now, it has been verified that some fungal endophytes isolated from medicinal plants have higher values of bioactive secondary metabolites than their hosts. A widely accepted definition of endophytes is that they are bacterial or fungal microorganisms growing in healthy plant tissues and not apparently harming their hosts (Stierle et al. 1993; Cao et al. 2005). Since the year of 1904, endophytes have been isolated from almost all host plants studied (Zhang et al. 2006; Sánchez Márquez et al. 2007; Huang et al. 2008). Endophytes have been recognized as potential sources of novel natural products for pharmaceutical, agricultural and industrial uses, especially the secondary metabolites produced by fungal endophytes colonizing medicinal plants (Hyde and Soytong 2008; Mitchell et al. 2008). However, the production of highly bioactive secondary metabolites reported from several endophytes is limited, such as *Taxus brevifolia* S.F Grey (Taxaceae) (Wani et al. 1971), *Gentiana macrophylla* Pall (sect. cruciata gaduin) (Gentianaceae) (Hong 2009), Icacinaceae (Shweta et al. 2010) and Celastraceae (Pullen et al. 2002).

Classical methods for strain improvement have been applied to successfully produce a number of industrial strains, but they are time-consuming and laborious due to the repeatedly random mutation and selection. Recently, an efficient technology named genome shuffling has made great progresses in the construction of mutants with distinctly and significantly improved phenotype. The tylosin production from *Streptomyces fradiae* has been rapidly reinforced by two rounds of genome shuffling, although 20 rounds of mutagenesis and screening were required in the past (Zhang et al. 2002). Genome shuffling allows many parental strains with certain phenotypic improvements to be recombined through recursive protoplast fusion. A library of shuffled bacteria with genetic exchange is achieved by repeating the above process. Since the limited knowledge about genome sequence negatively affects the rational application of recombinant DNA techniques to manipulate the strain, genome shuffling exhibits the advantage of recombination between genomes in uncharacterized organisms. This approach has also been used to improve the acid tolerance in *Lactobacillus* (Patnaik et al. 2002), degradation of pentachlorophenol in *Sphingobium chlorophenolicum* (Dai and Copley 2004) and production of hydroxycitric acid in *Streptomyces* (Hida et al. 2007). Moreover, our research group has already applied genome shuffling to improve the acid tolerance and volumetric productivity in *Lactobacillus rhamnosus* (Wang et al. 2007).

In this study, high-yield tanshinone IIA-producing strains were bred by genome shuffling. The protocols for isolating, regenerating protoplasts and conducting successive rounds of protoplast fusion in *Emericella foeniculicola* (*Eurotiales*) (Aspergillaceae *Aspergillus*) were described. Finally, a high-yield producer of tanshinone IIA was obtained, and its productivity was confirmed.

## Materials and methods

### Microorganisms

TR21 was isolated from *S. miltiorrhiza* in our previous study (Wei et al. 2010). The tanshinone IIA-producing strains U104, NU152, NU204 and NU256 which mutated from the original strain TR21 were maintained on PDA medium (Ma et al. 2011).

### Chemicals and reagents

Tanshinone IIA was purchased from the State Drug Administration (Shaanxi, China). Except for methanol (Tianjin Kermel Chemical Plant, of HPLC grade China), all other chemicals and reagents fell into the category of analytical grade and were purchased from commercial sources, unless otherwise stated.

### Endophytic fungus and culture conditions

One 5 mm fungal block was inoculated into a 50 mL PDB (potato dextrose broth) fermentation medium which was sterilized at 121 °C for 30 min in a 250 mL Erlenmeyer flask. Then the inoculum was cultured at 28 °C on an oscillating table at the rate of 160 rpm for 7 days.

### Genome shuffling

Genome shuffling was conducted with the aforementioned method (Zhang et al. 2002; Patnaik et al. 2002; Dai and Copley 2004; Hida et al. 2007) with some modifications. Samples of protoplasts (0.5 mL) were prepared by using hypertonic solution I (0.5 M KCl, 25 mM Tris–HCl buffer, pH 6.0) from two different strains which were mixed and equally divided into two aliquots. One aliquot was inactivated with UV light for 10 min, while the other one was heat treated at 50 °C for 50 min. Protoplasts inactivated by those two different methods were mixed, centrifuged, and resuspended in 1 mL of hypertonic solution II (0.5 M sucrose, 25 mM Tris–HCl buffer, pH 6.0). 9 mL of 30% PEG 4000 with 15% dimethyl sulfoxide (DMSO), and 10 mL of CaCl_2_ in buffer B was then added to the resuspended protoplast mixture and incubated for 7 min at 30 °C with the shaking speed of 100 rpm. The resultant fused protoplasts were centrifuged, washed for three times with hypertonic solution II, resuspended in 1 mL hypertonic solution II and then regenerated on regeneration medium plates in the incubator at 28 °C for 7 days. The fusion frequency was calculated as the ratio of the number of fusants from the protoplasts treated with PEG to the number of the regenerated strains without inactivation treatment. The large and high-yield tanshinone IIA-producing colonies were selected. Based on the screening and analysis methods described above, the best shuffled mutants were taken for the subsequent genome shuffling. The pooled fusion libraries were named as F1, F2 and F3, respectively.

### Extraction of cell-associated tanshinone IIA from cells

Strains were cultivated in 50 mL PDB at 28 °C for 7 days, and each fermentation broth was centrifuged at 5000 rpm for 30 min to obtain mycelium pellets. The mycelium pellets were then thoroughly washed with sterile distilled water, dried to constant weight at 70 °C, powdered in a glass mortar with a pestle, and extracted with 12 mL methanol by BILON88-II ultrasonic cell disintegrator. All the samples were filtered twice by disposable syringe filtering cartridges with 0.22 µm nylon membranes and detected by TU-1810 UV/VIS (ultraviolet–visible) spectrophotometer (Beijing, China) for a preliminary test. The samples of improved strains were analyzed by LC-2010A high-performance liquid chromatography (Shimadzu, Japan) for a more accurate test. The cell growth was monitored by measuring the weight of dried cells.

### High-performance liquid chromatography (HPLC) analysis

HPLC analysis was performed by using a Dikma Inertsil ODS chromatographic column (5 mm × 4.6 mm × 250 mm in size) which was adjusted to 30 °C with a column heater. The optimized absorption wavelength was selected as 270 nm after UV–visible absorption measurement of tanshinone IIA. The mobile phase was a mixture of methanol and water (85:15, v/v) which was filtered and degassed by a PTFE filter (4 mm × 0.45 mm in size). The flow rate was 0.8 mL/min and the total run time per injected sample was 30 min. For each sample, the injection volume was 10 µL and three injections were made to gain the averages and relative standard deviations were reported in this paper. The regression equation and correlation coefficient were A = 6.22 × 10^7^C-33147 and *R*^2^ = 0.9994 (*n* = 5), respectively, where A was the peak area of tanshinone IIA and C was the concentration of tanshinone IIA (mg per mL). The detector response was linear from 0.0064 to 0.064 mg per mL and was used to provide quantitative data. This method was sensitive, accurate and reproducible.

### RAPD analysis of the nuclear genomes

The total DNAs of parental and daughter strains were extracted from dry mycelia according to a modified CTAB procedure (Dai and Copley 2004). 20 pairs of RAPD primers selected randomly from 200 pairs (Shanghai Sangon Co., China Table 1) were used. RAPD amplification was conducted in a 25 µL reaction volume consisting of 10 × PCR buffer (100 mM Tris-HCl, pH 9.0, 500 mM KCl, 1% Triton-X100), 1.5 mM MgCl_2_, 200 M dNTPs, 1 unit Taq polymerase, 100 ng of genomic DNA template and 0.4 mM primer. PCR was performed in a PTC-100 Thermal Cycler (MJ Research) with an initial denaturation phase at 95 °C for 5 min, followed by 40 cycles at 95 °C for 1 min, 37 °C for 1.5 min, 72 °C for 3 min and a final extension at 72 °C for 10 min. PCR products were fractionated by electrophoresis using 1.2% agarose gels with ethidium bromide in 0.5 × TBE buffer at 100 V for 1 h and visualized by ultraviolet light.

**Table 1.** Primer sequences used for RAPD.

Primers	Sequence	Primers	Sequence
S61	TTCGAGCCAG	S62	GTGAGGCGTC
S63	GGGGGTCTTT	S64	CCGCATCTAC
S65	GATGACCGCC	S66	GAACGGACTC
S67	GTCCCGACGA	S68	TGGACCGGTG
S69	CTCACCGTCC	S70	TGTCTGGGTG
S71	AAAGCTGCGG	S72	TGTCATCCCC
S73	AAGCCTCGTC	S74	TGCGTGCTTG
S75	GACGGATCAG	S76	CACACTCCAG
S77	TTCCCCCCAG	S78	TGAGTGGGTG
S79	GTTGCCAGCC	S80	ACTTCGCCACA

### Statistical analysis

Each experiment was performed three times, and the results were expressed as mean ± SD. Statistical analysis was done by one-way repeated measurement analysis of variance. The statistical differences between the treatment group and the control group were determined by independent-samples *t-*test. *p < 0.05, that is, a significant difference from the control group, was presented in the figures.

## Results and discussion

### Genome shuffling to generate high-yield strains

Genome shuffling is dependent upon the recursive fusion of protoplasts to carry out recombination. This recursive strategy permits the quick obtainment of phenotypes of interest. The high frequency of protoplast formation and regeneration is the basis of the efficient genome shuffling. When the protoplasts were mixed with PEG solution, they stuck together and then formed paired protoplasts. Later, the plasma membranes in the contact area of protoplasts were dissolved, and the protoplasmic contents were fused. The fusion of two or more cell membranes and the resultant formation of a heterokaryon were observed through a high-power microscope. Subsequently, the nuclei of some paired protoplasts were fused together (karyogamy). Ultimately, the fused protoplasts were turned into single, large, round or oval-shaped structures, respectively (data not shown).

After the first fusion, six strains (F1) were obtained from the first shuffled library and used for the second fusion. Four colonies (F2) were obtained from the second shuffled library and used for the next fusion. After the third fusion, four colonies (F3) were obtained from the third shuffled library.

### Tanshinone IIA production of mutant stains

**Figure 1.**
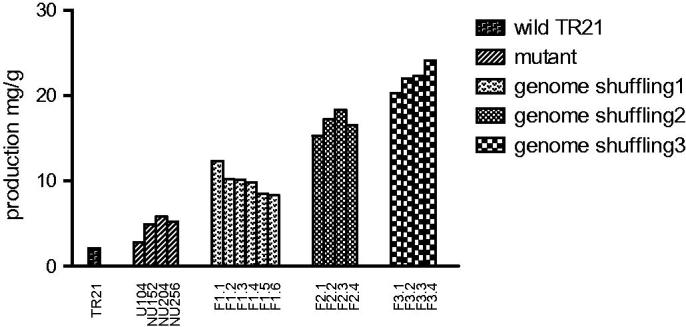
Improvement of tanshinone IIA production by genome shuffling.

To compare the production of tanshinone IIA between the wild strains and the mutant strains, the mutant strains were cultured in shake-flask containing PDB medium, then the cell-associated tanshinone IIA were extracted from cells. All the samples were detected by TU-1810 UV/Vis (ultraviolet–visible) spectrophotometer for a preliminary test. The result was shown in Figure 1. After three rounds of the genome shuffling, the strains production of tanshinone IIA was improved significantly. The tanshinone IIA was detected by TU-1810 UV/Vis spectrophotometer for a preliminary test. The highest-yield producer was F3-4, which could produce tanshinone IIA over 10 times more than the original strain TR21 did.

### Mutant second screening

**Figure 2.**
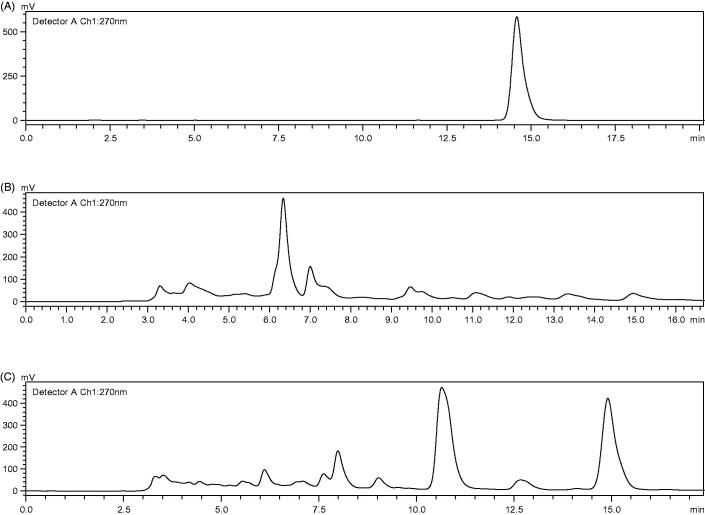
HPLC chromatograms of authentic compound tanshinone IIA (A), cell extract of TR21 (B) and cell extract of F3.4 (C). The ultrasonic extraction method had been developed for the extraction of tanshinone IIA from the fungi with analysis by HPLC.

The high-yield tanshinone IIA-producing strains were determined by high-performance liquid chromatography (HPLC). The HPLC results showed that the retention time of authentic tanshinone IIA was 14.797 min (Figure 2A). The retention time of extraction of cell-associated tanshinone IIA from TR21 was 14.872 min (Figure 2B) with the yield about 1.701 ± 0.34 mg/mL. The HPLC result (Figure 2C) showed that the retention time was 14.902 min and the content of tanshinone IIA produced by F3.4 had a relative high value, i.e. 18.827 ± 0.22 mg/mL. The F3.4 strains increased the yield of tanshinone IIA by more than 11.07 times, compared with the wild type one (*p* < 0.05). Then it was found that mutant strains which were generated by the genome shuffling mutagenesis had higher production yield of tanshinone IIA.

### Genetic stability of the fusion strains

The highest tanshinone IIA-producing strains F3.4 were transferred of culture for ten passages to ascertain its genetic stability, and it grew vigorously in every generation on PDA medium. The growth rate of strains in each generation was measured by weighing the dried mycelia harvested from culture on PDB liquid medium at the logarithmic phase. The cell density (OD 270 nm) of every generation was also measured. There was no apparent difference in the growth rate or the cell density composition among the different generations (*p* < 0.05, Table 2), which suggested that this hybrid was genetically stable and suitable for practical use.

**Table 2.** The genetic stability of F3.4.

Strains	Biomass (g/50 mL)	Production (mg/g)
F3.4-1	0.74 ± 0.06	6.24 ± 0.12
F3.4-2	0.73 ± 0.12	6.18 ± 0.06
F3.4-3	0.74 ± 0.06	6.22 ± 0.03
F3.4-4	0.71 ± 0.06	6.19 ± 0.03
F3.4-5	0.73 ± 0.03	6.15 ± 0.03
F3.4-6	0.69 ± 0.06	6.21 ± 0.03
F3.4-7	0.68 ± 0.06	6.18 ± 0.03
F3.4-8	0.71 ± 0.06	6.19 ± 0.03
F3.4-9	0.68 ± 0.06	6.17 ± 0.03
F3.4-10	0.69 ± 0.06	6.17 ± 0.03

### RAPD polymorphisms

**Figure 3.**
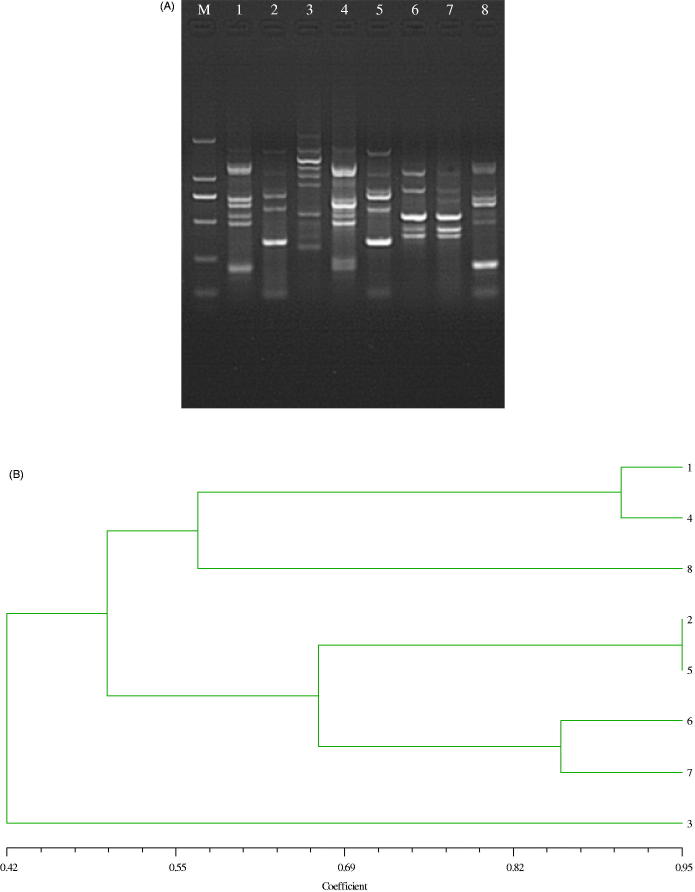
(A) Note: RAPD-PCR profiles of strains using primer S75. M is the DNA marker. Lanes 1–8 are the parent strain and the high-yielding isolates. (from left to right: TR21, F1.1, F2.3, F3.4, U104, NU152, NU204, NU256) by using primer S75. (B) Polymorphism of genomic population by RAPD analysis. Note: Dendrogram of the tanshinone IIA-producing mutants based on UPGMA cluster analysis and similarity index.

RAPD analysis was used to examine the genetic diversity among three shuffled recombinants with different tanshinone IIA productivity and parental strain. One of the prescreened primers (S75) successfully amplified the polymorphic DNA fragments. Amplified fragments were characterized based on their size, ranging from approximately 100–2000 kb (Figure 3A). Analysis by RAPD indicated that the genetic differences existed among the mutant strains, the shuffled strains and the original strains, which achieved the breeding purpose on the whole. RAPD band pattern was analyzed by the Nei similarity index, and a dendrogram was constructed based on the similarity matrix data by applying the UPGMA cluster analysis method (Figure 3B). The shuffled recombinants were segregated and high producers were clustered into a subgroup. Based on the dendrogram, close homology existed in those strains and the similarity coefficient of eight strains varied from 0.42 to 0.95. The shuffled recombinant F1.1 was closest to U104 (their genetic similarity coefficient was 0.95), but much higher than the high-yield strain F3.4. The specific bands and UPGMA dendrogram of cluster analysis obtained in our study showed that genetic information was transferred from the parental strains to the shuffling strains by genome shuffling and the genetic information of the shuffling strains was changed.

## Discussion

Genome shuffling has recently been introduced and applied as an efficient method of producing microbial strains with desirable phenotypes (Ming et al. 2005). This approach has been used to improve the production of the polyketide antibiotic tylosin in *Streptomyces fradiae* (Zhang et al. 2002) and the acid tolerance of *Lactobacillus* (Patnaik et al. 2002).

In this study, a genome shuffling protocol for isolating the regenerating protoplasts and the successive rounds of protoplast fusion in *E. foeniculicola* was described. Our results demonstrate that the rapid improvement of tanshinone IIA production could be achieved by this method. After three rounds of protoplast fusion, an improved recombinant F3.4 was obtained. The molecular differences between the parental strains and the shuffled recombinants were observed. Our results suggest that strain F3.4 is a promising candidate strain for industrial application, and the genome shuffling is a powerful approach for the molecular breeding of industrial strains.
